# Supplementary material for: The Potential of Caffeic Acid Lipid Nanoparticulate Systems for Skin Application: In Vitro Assays to Assess Delivery and Antioxidant Effect
Source: Nanomaterials (Basel). 2021 Jan 12;11(1):171. doi: 10.3390/nano11010171 (PMC7826983; doi:10.3390/nano11010171)
Supplement: Supplementary file 1 [file nanomaterials-11-00171-s001.pdf]

Supplementary material

The potential of caffeic acid lipid nanoparticulate systems for skin application: in vitro assays to assess delivery and antioxidant effect

Supandeep Singh Hallan [1,2], Maddalena Sguizzato [1], Markus Drechsler [3], Paolo Mariani [4], Leda Montesi [5], Rita Cortesi [1,*], Sebastian Björklund [2], Tautgirdas Ruzgas [2,*] and Elisabetta Esposito [1,*]

$$2H_2O_2 \xrightarrow{Cat} 2H_2O + O_2 \tag{S1}$$

$$2H_2O_2 + CA \xrightarrow{Peroxidase\ like\ enzymes\ in\ skin} 2H_2O + CA_{ox} \tag{S2}$$

Equations S1 and S2 represent well-known reactions catalyzed by enzymes, such as catalase and peroxidase, respectively. It is important to note that catalase reaction produce O_2 which can be easily monitored by oxygen electrode. In peroxidase type reactions polyphenols are oxidized by H_2O_2. In this study CA (caffeic acid) acts as a polyphenolic substrate. There are a high number of enzymes that can catalyze peroxidase-like reactions (see H.B. Dunford, Peroxidases and Catalases: Biochemistry, Biophysics, Biotechnology and Physiology, 2nd

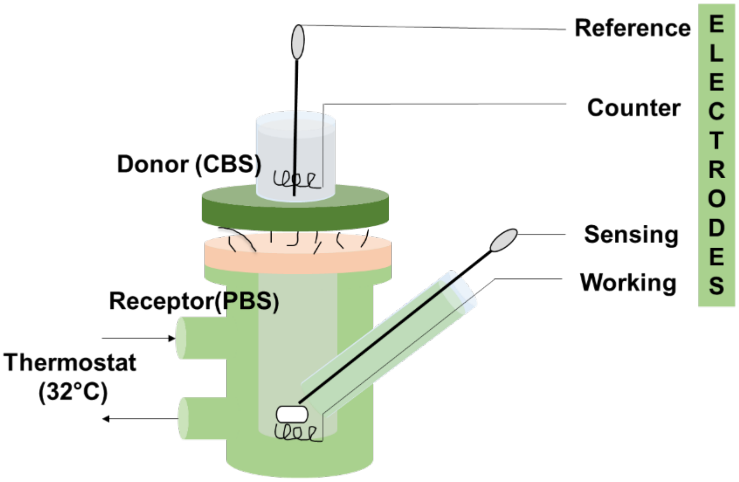

ed., Wiley-Blackwell, New Jersey, 2010).

Figure S1. Franz diffusion cell (PermeGear Inc. Hellertown, PA, USA) equipped with 4 electrodes for skin membrane resistance measurements using electrical impedance spectroscopy. In brief, an electrical current that passes the skin membrane is generated between the counter and the working electrodes. The reference and sensing electrodes measure the potential drop over the membrane and the solution enclosed between these electrodes. By using Ohms law, the impedance of the skin membrane and the enclosed solution is calculated as a ratio of the potential drop to the passing electrical current. The measurements and the calculations are done automatically by connecting the electrodes to Ivium potentiostate (Ivium Technologies, Eindhoven, Netherlands) and exploiting Ivium software, respectively. More comprehensive description of the Franz cell, equipped with electrodes, and the methodology of impedance measurements can be found in our previous publications (S. Björklund, T. Ruzgas, A. Nowacka, I. Dahi, D. Topgaard, E. Sparr, and J. Engblom. Skin membrane electrical impedance properties under the influence of a varying water gradient. Biophys. J. (2013), 104, 2639-2650.).

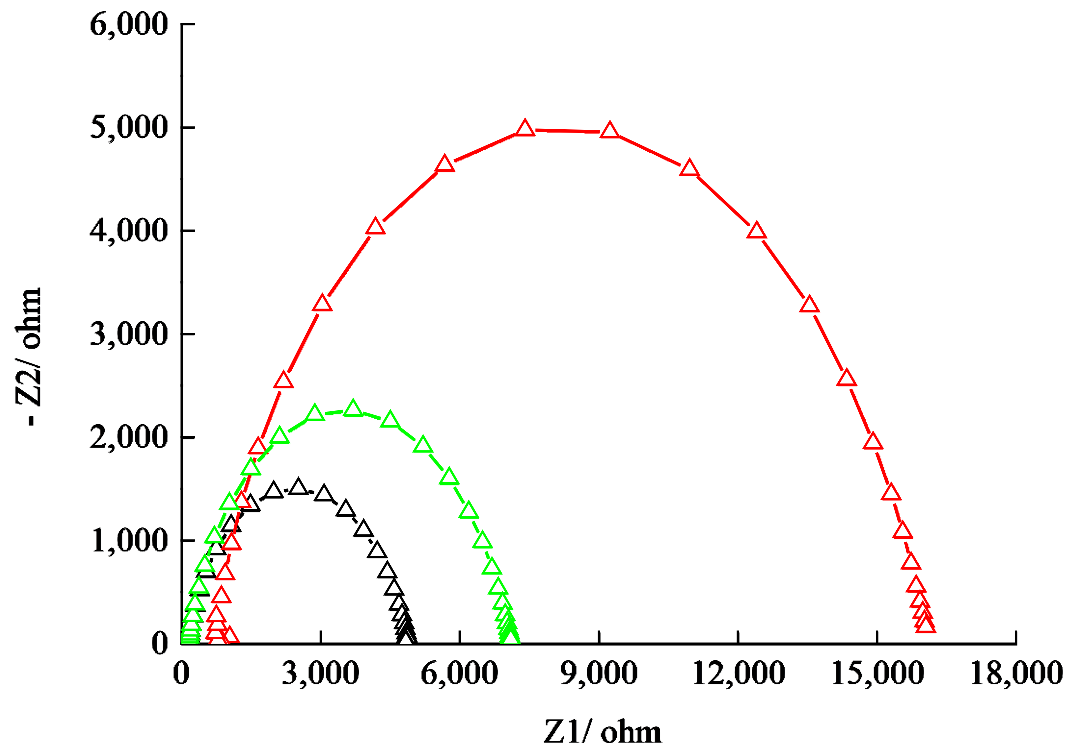

Figure S2: An example of impedance data in Nyquist coordinates obtained for skin membrane in CBS in the absence and in the presence of nanoparticle formulation. The data were recorded using the Franz cell equipped with 4 electrodes described in Figure S1. Skin resistance values were determined by fitting the of data to the impedance of the equivalent circuit presented in Figure S3 using Ivium software.

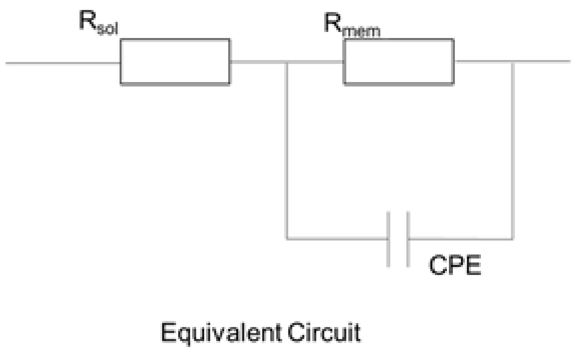

Figure S3: Electrical equivalent circuit modelling the impedance of a skin membrane in the Franz cell as shown in Figure S1. R_{sol} represents resistance of solutions surrounding the membrane. R_{mem} represents skin membrane resistance. CPE is a constant phase element and represents capacitive properties of skin membrane. More comprehensive explanation of skin impedance and the equivalent circuits that describe impedance properties if skin membranes can be found in our previous publication: S. Björklund, T. Ruzgas, A. Nowacka, I. Dahi, D. Topgaard, E. Sparr, and J. Engblom. Skin membrane electrical impedance properties under the influence of a varying water gradient. Biophys. J. (2013), 104, 2639-2650.

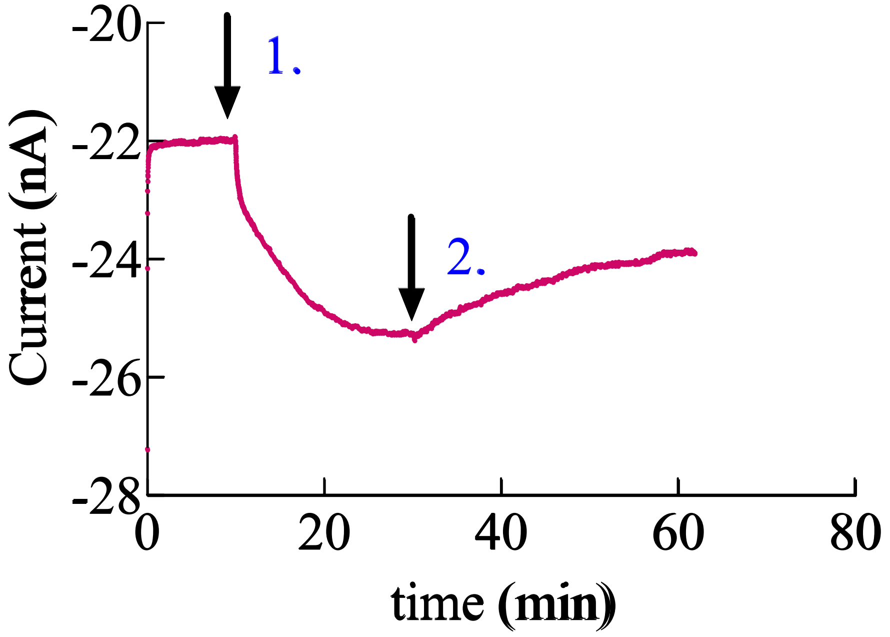

Figure S4: A typical current response of skin membrane covered oxygen electrode (SCOE) to (1) 0.5 mM H_2O_2 and (2) 0.5 mM of CA. The SCOE is in CBS solution and the mentioned compounds are added into the solution at times indicated by arrows. The solution is stirred by magnetic stirrer. The current trace of the electrode before "1" is due to the dissolved oxygen present in CBS. After addition of H_2O_2, the H_2O_2 penetrates the skin membrane, which is firmly fixed at the tip of the oxygen electrode. Catalase, present in the skin, converts H_2O_2 to water in oxygen (see equation 1s). This increase of oxygen concentration in skin (placed right on the tip of the oxygen electrode) and gives an increase in the reduction current of the electrode (reduction current is noted with minus sign by the definition). When a steady-state between the H_2O_2 diffusion (H_2O_2 supply into the skin) and the catalase reaction (H_2O_2 consumption in the skin) is reached, the current levels-off. This is observed right before "2". At this moment (marked with arrow "2") caffeic acid is added into the solution. This polyphenol penetrates the skin membrane and is oxidized by H_2O_2 catalyzed by peroxidase-like enzymes present in skin (see equation 2s). These peroxidase-like reactions remove some part of H_2O_2 from the catalase reaction and thus result in decreased O_2 concentration in skin. The process is monitored as a decrease in the current of the oxygen electrode (the current trace after arrow marked with "2"). More comprehensive description of the SCOE construction and the methodology can be found in our previous publications. (a) M. Eskandari, J. Rembiesa, L. Startaitė, A. Holefors, A. Valančiūtė, F. Faridbod, M. R. Ganjali, J. Engblom, T. Ruzgas. Polyphenol-Hydrogen Peroxide Reactions in Skin: In vitro Model Relevant to Study ROS Reactions at Inflammation. Anal. Chim. Acta. 1075 (2019) 91-97; (b) S. Nocchi, S. Björklund, B. Svensson, J. Engblom, and T. Ruzgas. Electrochemical monitoring of native catalase activity in skin using skin covered oxygen electrode. Biosens. Bioelectron. 93 (2017) 9-13.

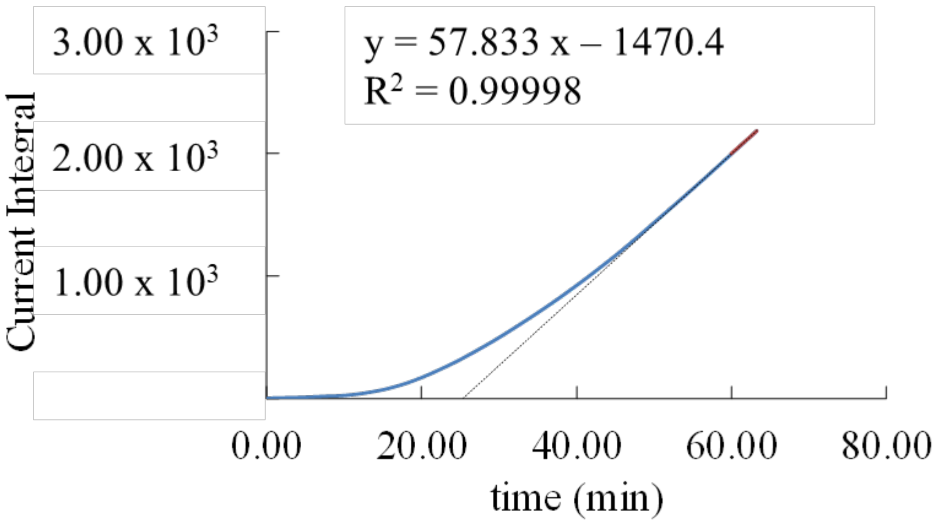

Figure S5: An example of a plot of integral of current response of SCOE vs time reconstructed from an original current response measurement with SCOE as shown in Figure S4. These types of plots were used to estimate a lag time for calculation of apparent diffusion coefficients. Time zero in these plots is assigned to the moment when an appropriate compound or a formulation of interest was added into the measurement cell. The line in this plot represents a fitting of a part of the data where the linear dependence of the current integral vs time is observed. The comprehensive explanation of the approach can be found in our earlier publications: (a) S. Ullah, F. Hamade, U. Bubniene, J. Engblom, A. Ramanavicius, A. Ramanaviciene, T. Ruzgas. In-vitro model for assessing glucose diffusion through skin. Biosens. Bioelectron. 110 (2018) 175-179. (b) M. Eskandari, J. Rembiesa, L. Startaitė, A. Holefors, A. Valančiūtė, F. Faridbod, M. R. Ganjali, J. Engblom, T. Ruzgas. Polyphenol-Hydrogen Peroxide Reactions in Skin: In vitro Model Relevant to Study ROS Reactions at Inflammation. Anal. Chim. Acta. 1075 (2019) 91-97.
